# Fiber Coupled Transceiver with 6.5 THz Bandwidth for Terahertz Time-Domain Spectroscopy in Reflection Geometry

**DOI:** 10.3390/s20092616

**Published:** 2020-05-04

**Authors:** Robert B. Kohlhaas, Lars Liebermeister, Steffen Breuer, Marcel Amberg, David de Felipe, Simon Nellen, Martin Schell, Björn Globisch

**Affiliations:** 1 Fraunhofer Institute for Telecommunications, Heinrich Hertz Institute, Einsteinufer 37, 10587 Berlin, Germany; lars.liebermeister@hhi.fraunhofer.de (L.L.); steffen.breuer@hhi.fraunhofer.de (S.B.); marcel.amberg@hhi.fraunhofer.de (M.A.); david.felipe@hhi.fraunhofer.de (D.d.F.); simon.nellen@hhi.fraunhofer.de (S.N.); martin.schell@hhi.fraunhofer.de (M.S.); bjoern.globisch@hhi.fraunhofer.de (B.G.); 2 Institut für Festkörperphysik, Technische Universität Berlin, Hardenbergstraße 36, EW 5-1, 10623 Berlin, Germany

**Keywords:** THz time-domain spectroscopy, photoconductive antennas, THz transceiver, THz imaging, non-destructive testing

## Abstract

We present a fiber coupled transceiver head for terahertz (THz) time-domain reflection measurements. The monolithically integrated transceiver chip is based on iron (Fe) doped In_0.53_Ga_0.47_As (InGaAs:Fe) grown by molecular beam epitaxy. Due to its ultrashort electron lifetime and high mobility, InGaAs:Fe is very well suited as both THz emitter and receiver. A record THz bandwidth of 6.5 THz and a peak dynamic range of up to 75 dB are achieved. In addition, we present THz imaging in reflection geometry with a spatial resolution as good as 130 µm. Hence, this THz transceiver is a promising device for industrial THz sensing applications.

## 1. Introduction

The terahertz (THz) spectral range (0.1–10 THz) combines a diverse range of fundamental research questions and technically useful properties. For example, many materials of industrial and technical importance, such as ceramics, paper, (functional) coatings, automotive paint, or plastics are transparent in the THz range [1,2,3]. This allows non-contact, non-destructive testing including defect identification/localization and the determination of layer thicknesses, even of multi-layered samples [4,5,6]. THz time-domain spectroscopy (TDS), which uses pulsed THz radiation generated by ultrashort optical pulses, has become an established technique to address these applications [7,8]. In THz TDS, time-of-flight measurements are used to determine layer thicknesses of dielectric coatings with micrometer precision [6,9].

Due to the availability of mature and cost-efficient optical components and lasers at the telecommunication wavelength around 1550 nm, many commercially available THz systems exploit this technology. This enables all fiber-coupled THz TDS systems, which can be directly employed in industrial environments. The best-performing systems achieve a THz bandwidth of > 6 THz and up to 105 dB peak dynamic range [10,11].

Many industrial applications of THz TDS require measurements in reflection geometry since the device under test (DUT) is accessible from one side only, or since the substrate below the layer of interest is not transparent for THz radiation. Until today, most commercially available THz TDS systems use separate THz emitter and detector modules, which complicates reflection measurements significantly. In general, two configurations are employed: if normal incidence is needed, a THz beam splitter is used to guide the THz radiation reflected from the DUT to the receiver. The drawback of this approach is the high loss, which reduces the THz power at the receiver to at most 25% of the initially emitted power. In another prominent configuration, THz emitter and receiver are slightly tilted with respect to the surface normal of the DUT. Although the losses are significantly lower compared to the beam splitter solution, the angled THz beam path complicates the alignment of the sensor head. Hence, a THz transceiver (TRX), combining THz emitter and detector in a single device, is highly desirable. Furthermore, a THz TRX containing emitter and receiver on a single chip simplifies miniaturization of the THz sensor head, which may unlock many more applications for THz TDS.

For the development of an integrated THz transceiver, two challenges stand out: first, a monolithically integrated THz transceiver has to employ the same photoconductor for THz emission and detection. Since the requirements on the properties of the photoconductor are different for emitters and receivers, hitherto different photoconductive materials have been developed and optimized, respectively [12,13]. Second, the optical fiber coupling of a THz TRX is more challenging compared to a single emitter or receiver since two photoconductive gaps in a distance of a few tens of micrometers have to be illuminated independently of each other. In a previous publication we addressed the second challenge by developing a polymer waveguide-chip with low insertion loss, which enables the illumination of two photoconductive gaps with a predefined spacing [14]. In this publication, we focus on the first challenge and present a monolithically integrated THz transceiver based on iron (Fe) doped In_0.53_Ga_0.47_As (InGaAs:Fe) [15]. This photoconductor is very well suited for both THz emission and detection. We compare different transceiver designs with a varied photoconductive gap size and demonstrate a compact, fiber-coupled THz transceiver head with 6.5 THz bandwidth and a peak dynamic range up to 75 dB as the optimum. In addition, we compare the performance of this transceiver with a terahertz reflection head consisting of a state-of-the-art individual emitter and receiver module. Finally, we use the TRX for THz reflection imaging. Due to the confocal geometry, an unprecedented lateral resolution of 130 µm is obtained. These results underline that the THz TRX, which is fully compatible with commercially available THz TDS systems, is a versatile tool to address real-world applications.

## 2. Materials and Methods

### 2.1. Material Properties of Fe Doped InGaAs

In this section, the material properties of the InGaAs:Fe photoconductor, which is used for the monolithically integrated THz TRX, are discussed and compared to commonly used photoconductors. Note that generally the requirements on the photoconductor are different for THz emission and detection. An emitter requires above all a high mobility and large breakdown field strength in order to accelerate optically excited carriers efficiently in a large bias field [16]. For THz receivers, a short, sub-picosecond electron lifetime is most important in order to sample the THz pulse accurately in the time-domain [16]. In addition to this short lifetime, a high mobility and high resistivity are very desirable to achieve a good responsivity and low noise level for the receiver. Satisfying the demands of the emitter and receiver at the same time complicates the development of a photoconductor for THz transceivers.

The major challenge in material development for THz antennas is the trade-off between short electron lifetime and high mobility. In general, point defects are introduced into the photoconductor in order to create fast trapping centers. However, these point defects also act as scattering centers for excited carriers, and therefore reduce the carrier mobility. Within the last three decades, different approaches such as ion implantation [17,18], low-temperature-growth (LTG) [13,19], erbium arsenide (ErAs) clusters [11], and different deep-level dopants have been investigated to generate trapping centers. The electrical and optical properties of the most prominent photoconductors are summarized in Table 1. Recently, we started to investigate transition metal doped InGaAs grown by molecular beam epitaxy (MBE) for THz emission and detection. With InGaAs:Fe we demonstrated that THz emitters and receivers fabricated from this material are comparable to the best state-of-the-art antennas based on different, individually optimized photoconductors [15]. Therefore, InGaAs:Fe promised to be an excellent material for the development of a THz TRX.

In this work, we employed InGaAs:Fe grown by gas-source MBE (Riber Compact 21 system) at a growth temperature of 325 °C. The layer stack consists of a Fe doped InGaAs layer (1200 nm thickness) on top of an In_0.52_Al_0.48_As buffer layer (700 nm thickness) grown lattice matched to a semi-insulating InP:Fe substrate. The Fe doping concentration of the InGaAs layer is > 1 × 10^20^ cm^−3^ as determined by secondary ion mass spectroscopy. A comparison of the properties of InGaAs:Fe with some other, established photoconductors is shown in Table 1.

**Figure 1:**
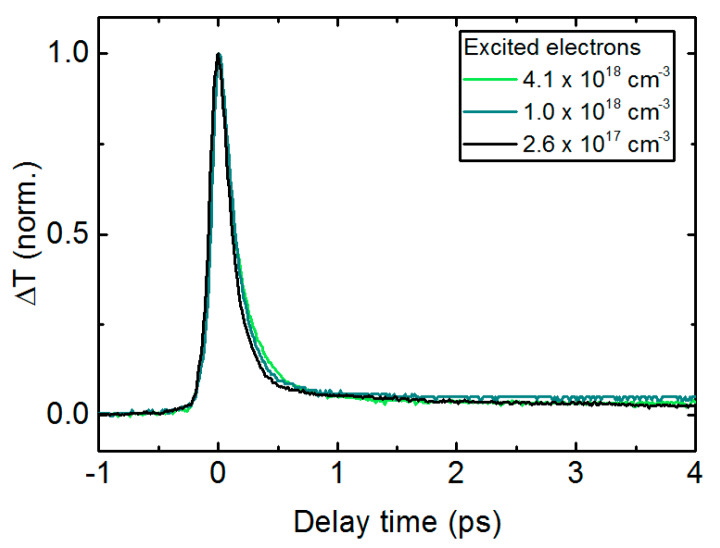
Differential transmission curves of InGaAs:Fe as a function of pump-probe time delay for three different electron densities excited by the 1550 nm pump. InGaAs:Fe features an ultrashort electron lifetime of 250 fs and shows no saturation of the electron lifetime for increasing optical excitation densities.

The electron lifetime was measured by wavelength degenerate optical pump-probe measurements at 1550 nm using an erbium doped fiber laser emitting 90 fs pulses at a repetition rate of 100 MHz [20]. The differential transmission curves for three different optical excitation densities are shown in Figure 1. Note that the carrier lifetime is as short as 250 fs and does not show pronounced saturation even for high excitation densities. This ultrafast carrier lifetime in InGaAs:Fe is a prerequisite for broadband THz detection.

In contrast, a commonly used material system for THz emitters only, an InGaAs/InAlAs superlattice (abbreviated as SL in Table 1) [12], is optimized for high mobility and high resistivity at the cost of a significantly longer electron lifetime around 40 ps. The LTG-InGaAs:Be photoconductor, used for THz receivers only, features an ultra-short electron lifetime (280 fs) at the expense of a reduced mobility and resistivity. In comparison to the photoconductors based on ErAs clusters, InGaAs:Fe shows an excellent combination of all desired parameters: a very short lifetime of only 250 fs, a large Hall mobility of 710 cm^2^/Vs, and a resistivity of 1570 Ω·cm. This makes InGaAs:Fe very well suited for application as a transceiver.

### 2.2. Fiber-Coupled THz Transceiver Sensor Head

**Figure 2:**
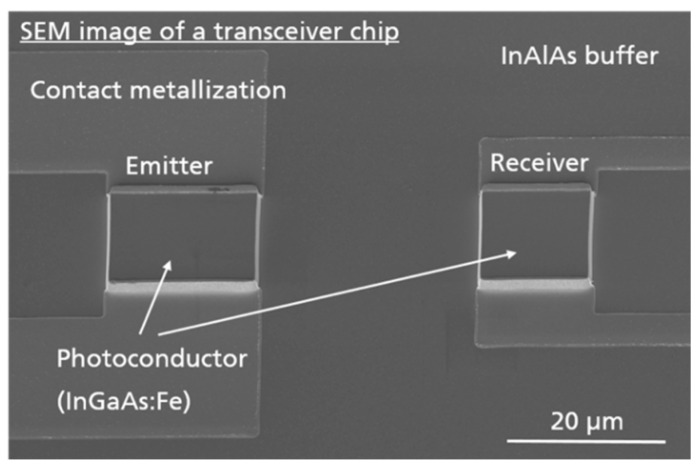
Scanning electron micrograph of a monolithically integrated THz transceiver chip. The emitter (strip-line antenna with a gap size of 15 µm) is located on the left, the receiver (also strip-line antenna with 15 µm gap width) on the right. The midpoints of the two photoconductive mesas lie only 45 µm apart.

A scanning electron micrograph of a typical transceiver chip is shown in Figure 2. The monolithically integrated THz transceiver chip consists of two separate photoconductive gaps. The center points of the emitter and receiver are 45 µm apart. The emitter is shown on the left, the receiver on the right. Both emitter and receiver antennas are processed as strip-line antennas with a gap size of 15 µm. Note that emitter and receiver are structured as mesas [21]. The THz transceivers investigated in this work are all based on InGaAs:Fe [22].

Instead of two separate photoconductive gaps as presented here, it is possible to design a transceiver with only a single, shared gap for emitter and receiver [23,24]. While this simplifies the optical coupling of the transceiver, the cross-talk and noise floor is increased significantly. Furthermore, it is difficult to extract a small THz signal while a large bias voltage is applied for emitter operation. Consequently, these earlier studies investigating transceiver designs with a single photoconductive gap were able to show only a limited bandwidth of around 1 THz.

For characterization, the transceiver chips were packaged into fiber-coupled THz modules. First, the transceiver chip was positioned in the center of a hyperhemispherical silicon lens with a diameter of 10 mm. Electrical contacts were realized via wire bonding to PCBs. For the optical fiber coupling, a polymer waveguide chip, which allows for the independent illumination of the two photoconductive gaps, was used in analogy to the procedure described in [14]. THz pulse traces were recorded with a commercially available time-domain system employing an erbium doped fiber laser emitting optical pulses with a pulse width of 100 fs at 1550 nm central wavelength with a repetition rate of 100 MHz [25]. All measurements were done in ambient air. The total length of the THz beam path was 20 cm. Unless stated otherwise, the THz spectra shown in this publication were obtained by a FFT of THz pulse traces with 70 ps length. THz power spectra are normalized to the high frequency noise floor >7 THz. When the dynamic range is shown, it was calculated by dividing the power spectra by the noise spectra, which were obtained by blocking the THz path after subtracting the static background. Each pulse trace is an average of 1000 individual THz pulse traces that were acquired within a measurement time of 60 s. THz emitters were biased with an electric field of 40 kV/cm and the average optical power at both emitter and receiver was 20 mW.

## 3. Results and Discussion

In this section, the transceivers are characterized as sensor heads in THz TDS measurements in reflection geometry. In Section 3.1, we investigate THz transceiver chips with varying gap sizes in order to determine the optimal combination for high bandwidth emission and high dynamic range. Furthermore, the performance of the THz TRX is compared with a THz reflection head consisting of separate, state-of-the art emitter and receiver modules. In Section 3.2, the TRX is employed for THz imaging experiments. Due to the confocal geometry of the THz transceiver, the focal length of the parabolic mirror used for focusing the THz radiation on the DUT can be changed easily in order to increase the lateral resolution of the image.

### 3.1. Characterization of the THz Transceiver

**Figure 3:**
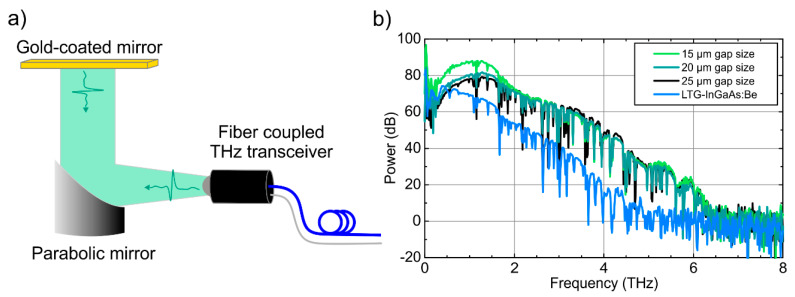
(**a**) Schematic of the setup used for THz reflection measurements with a fiber coupled THz transceiver. (**b**) THz spectra of different transceiver antennas measured in reflection geometry. To obtain equal optical excitation densities for TX and RX, equal photoconductive gap sizes were chosen, i.e., 15 µm (light green), 20 µm (dark green), and 25 µm (black). For comparison, the THz spectrum of a LTG-InGaAs:Be based transceiver is shown in blue (cf. [14]).

In this section, THz transceivers with three different photoconductive gap sizes are compared. In order to facilitate the optical coupling of the transceiver chip inside the sensor head, the gap size of the emitter and the receiver part of the TRX were chosen to be equal (cf. Figure 2). In Figure 3a, the measurement setup that was used for all THz TDS measurements in reflection geometry with fiber-coupled THz transceiver modules is shown: the emitted radiation, which is divergent with an angle of ±15°, is collimated by a parabolic mirror with 3″ focal length and reflected from a planar, gold-coated mirror back onto the transceiver. Figure 3b compares the THz spectra for InGaAs:Fe based transceivers with a gap size of 15 µm (light green), 20 µm (dark green), and 25 µm (black), respectively. Note that all InGaAs:Fe based transceivers exhibit a bandwidth of more than 6 THz and a peak spectral power ≥80 dB in comparison to the high frequency noise floor >7 THz. This is a significant improvement compared to the best transceiver results published so far: in [14], a similar, monolithically integrated THz transceiver based on low-temperature-grown, Be doped InGaAs was presented. This LTG-InGaAs:Be based transceiver achieved a bandwidth of 4.5 THz and a peak power of 70 dB. For comparison, the THz spectrum of such a transceiver is shown in Figure 3b in blue. Thus, the InGaAs:Fe TRX presented here increases the bandwidth and spectral power by more than 1.5 THz and up to 20 dB, respectively. These improvements stem from the superior electrical and dynamic properties of the InGaAs:Fe photoconductive material as discussed in Section 2.1.

As shown in Figure 3b, the difference between 20 and 25 µm wide gaps is relatively small, whereas a significant increase (≈10 dB) in spectral power is obtained for the smallest employed gap size of 15 µm. For this antenna geometry, a bandwidth of 6.5 THz in combination with a normalized spectral power of 90 dB at 1 THz is achieved.

**Figure 4:**
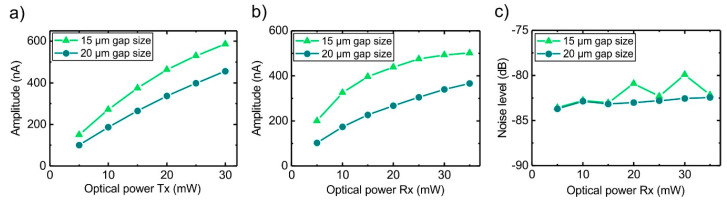
Characterization of the emitter and receiver part of the THz transceiver for 15 and 20 µm wide photoconductive gaps. (**a**) The emitted THz pulse amplitude as a function of optical power at the emitter, detected by a separate state-of-the-art THz receiver. (**b**) The detected THz pulse amplitudes emitted from a state-of-the-art emitter and (**c**) the spectral noise level for frequency components >7 THz as a function of optical power at the receiver part. As shown, the smaller photoconductive gap leads to higher emitted and detected THz pulse amplitudes while the spectral noise floor remains as low as for the larger gap size.

In order to analyze the properties of the presented THz transceiver further, we characterized emitter and receiver part of the TRX separately. First, we studied the emitted THz pulse amplitudes of the emitter part detected by a separate, state-of-the-art LTG-InGaAs:Be/InAlAs:Be based THz receiver. As shown in Figure 4a, the emitted amplitude as a function of optical power is higher for the TRX with the 15 µm wide gap versus 20 µm gap size. Furthermore, as Figure 4b shows, the detected THz pulse amplitudes are also larger for the smaller photoconductive gap. Since the noise level is approximately equal for the two gap sizes (cf. Figure 4c), the transceiver with the smaller gap size shows the overall best performance (cf. Figure 3b). Since the applied bias field was kept constant at 40 kV/cm for the emitter, we attribute the higher emitted THz pulse amplitudes to the higher carrier density induced by the optical excitation due to the smaller gap size. For the receiver part, we attribute the higher detected THz pulse amplitudes to the higher induced carrier density and also to a higher induced field in the antenna for a given incoming THz field strength. Both carrier density and induced field strength are increased due to the smaller photoconductive gap size.

**Figure 5:**
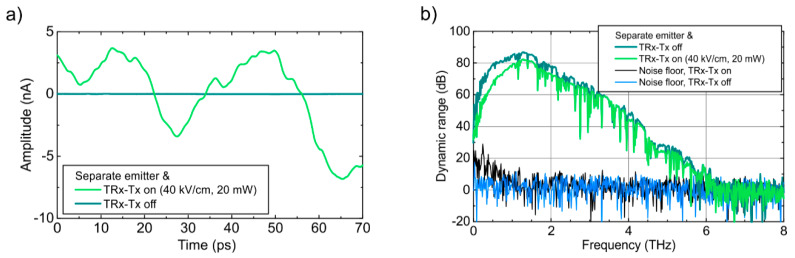
Influence of the emitter part of a THz transceiver on the detected THz signal emitted by a separate THz emitter. (**a**) The detected time-domain signals when the THz path is blocked and (**b**) the THz power spectra for a deactivated emitter part (dark green) vs. an activated emitter part at 40 kV/cm bias and 20 mW optical power (light green). The corresponding noise levels are shown in blue and black, respectively.

Second, since the midpoints of the TX and RX photoconductive gaps on the TRX chip are separated by 45 µm only (see Figure 2), we investigated the influence of an active emitter part on the performance of the receiver part of the TRX. For these measurements, we split the optical beam leading to the emitter part of the TRX into two arms with equal optical power. While one of these arms kept exciting the emitter part of the TRX, the other optical beam was used to drive a separate, fiber coupled emitter. In this configuration, we can characterize the receiver part of the TRX with the separate emitter, while the emitter part of the TRX could be switched on and off. Figure 5a shows the time-domain signal detected by the receiver part, when the separate emitter was switched off and the emitter part of the TRX was on (light green) or off (dark green). As can be seen, the active emitter part leads to a pronounced background in the receiver signal, which is caused by direct cross-talk between the emitter and the receiver through the substrate of the TRX chip. However, the peak-to-peak amplitude of this background is less than 5% of the THz pulse amplitudes shown in Figure 4a,b. In addition, the background is static and, therefore, it can be subtracted from the measurement signal. This procedure is applied for the measurements shown in Figure 5b: first, the THz path between the separate emitter and the TRX receiver was blocked in order to record the background when the emitter part is switched on and off. Next, this background was subtracted and the noise floor was determined by measuring the same configuration with a blocked THz path again. In Figure 5b, the noise floor for the emitter part switched on (black) and off (blue) is shown. As can be seen, the spectral noise floor of the TRX with the emitter part switched on (black) is spectrally flat between 1.5 and 8 THz. Hence, the dynamic range in in this frequency range remains almost as high as for the deactivated TRX emitter part. Below 1.5 THz, an activated TRX emitter causes excess noise reducing the dynamic range in this range. The dynamic range is calculated by dividing the power spectra by the noise spectra, which were obtained by blocking the THz path after subtracting the static background.

**Figure 6:**
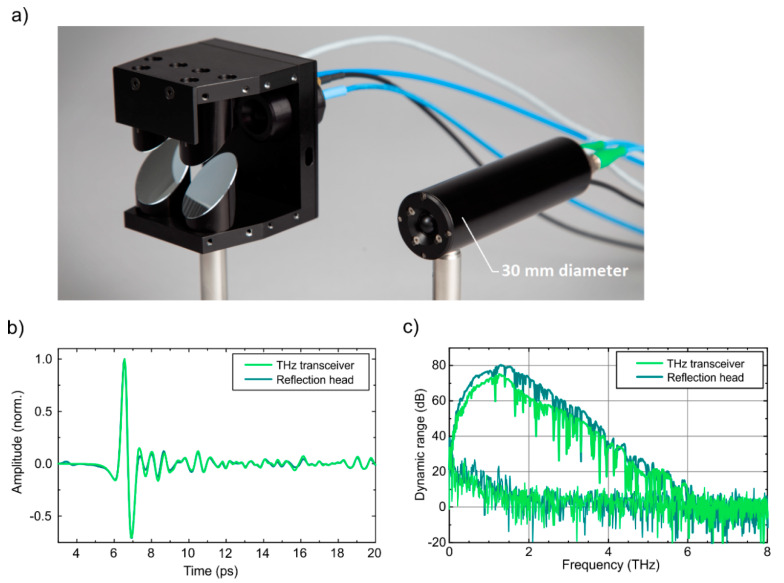
Comparison of THz reflection measurements for a reflection head with separate THz modules and angled beam path versus the integrated THz transceiver module. (**a**) A photograph of the (open) reflection head including its four parabolic mirrors and the fiber coupled THz transceiver module. In (**b**), the normalized THz pulse traces are compared, and (**c**) shows the corresponding dynamic range obtained by dividing the power spectrum by the spectral noise from a blocked THz path.

Finally, we compare the transceiver module with a reflection head consisting of an individual, state-of-the-art InGaAs:Fe based THz emitter and a LTG-InGaAs:Be/InAlAs:Be based receiver module. Figure 6a shows a photograph of the reflection head next to the fiber coupled transceiver module. The reflection head uses four parabolic mirrors to collimate the emitted THz radiation, focus it onto the sample and redirect the reflected pulse toward the THz receiver. Thus, the THz path within this reflection head is angled. Note that with a diameter of only 30 mm the fiber coupled transceiver module is significantly smaller than the reflection head.

In Figure 6b the normalized pulse traces detected with the reflection head (dark green) and the transceiver (light green) are compared. The shape of the two pulse traces is almost identical although the reflection head uses two individual THz modules and has an angled THz beam path, while emitter and receiver are integrated in very close proximity to each other on the TRX chip. This is mainly because InGaAs:Fe photoconductors from the same wafer were used for the individual THz modules and the THz transceiver. In addition, transceiver and reflection head were operated under the same excitation conditions with the same THz TDS system. Hence, Figure 6b clearly demonstrates that the integrated transceiver does not have any detrimental effect on the THz pulse trace. The small differences between the two pulse traces are attributed to differing optical coupling schemes inside the fiber-coupled modules leading to slightly different optical excitation powers in the reflection head and the transceiver, respectively.

Figure 6c shows the corresponding dynamic range over the whole THz spectrum. The dynamic range is calculated by dividing the power spectra by the noise spectra, which were obtained by blocking the THz path after subtracting the static background. Both reflection head and transceiver show a THz bandwidth of >6 THz. The peak dynamic range of the reflection head is 80 dB at 1.25 THz, while the transceiver reaches 75 dB peak DNR at 1.25 THz. Note that this is a record-high value for integrated THz transceivers. In comparison to the LTG-InGaAs:Be based transceiver presented in [14], the TRX presented in this paper features a 20 dB higher dynamic range and a 1.5 THz higher bandwidth. In comparison to the reflection head, the dynamic range of the transceiver is only 5 dB lower. In addition to the increased cross-talk between emitter and receiver, which has been discussed above, the single chip transceiver requires another important compromise: for individual antennas, the best performance could be achieved when the photoconductive gap of the emitter is larger (25 µm wide) than the gap of the receiver (10 µm wide). However, the optical coupling scheme with the polymer waveguide chip in the TRX creates optical spot sizes with equal diameters. Hence, equal gap sizes have to be used for emitter and receiver on the TRX chip, which reduces the performance of the TRX slightly. In addition, the simultaneous alignment of two photoconductive gaps in the TRX is more challenging than aligning a single gap for individual emitters and receivers. Regardless of all these challenges in the design and packaging of a fiber-coupled transceiver head, the performance of the TRX is quite close to the reflection head. In addition, the transceiver benefits from its collinear THz beam path and its small footprint, which makes it a perfect tool for reflection measurements in industrial environments. In the next paragraph, we will show how an integrated transceiver with collinear geometry can facilitate terahertz imaging experiments significantly.

### 3.2. THz Imaging with a THz Transceiver Module

Many interesting samples for THz imaging allow measurements in reflection geometry only. Without employing advanced near-field techniques to break the diffraction limit, the lateral resolution of THz images is inherently limited by the relatively long wavelengths corresponding to the THz frequency range: in air, the frequencies between 100 GHz and 5 THz correspond to wavelengths of 3 mm to 60 µm.

Here, we employ the fiber-coupled THz transceiver in a THz imaging setup in reflection geometry and demonstrate an improved lateral resolution compared to the reflection head with separate THz modules and angled beam path. The benefit of the integrated transceiver for this measurement is its collinear geometry, which means that only two parabolic mirrors are needed for measurements in reflection geometry: the first mirror collimates the emitted radiation; the second focusses it on the sample, from where the THz pulses are reflected back onto the receiver part of the TRX. Thus, in contrast to two individual THz modules, no angled beam path or beam splitter is necessary. Furthermore, the collinear geometry of the transceiver allows for changing the focal length of the focusing parabolic mirror without major changes of the THz beam path. Thereby, the lateral resolution of the image can be increased due to a smaller beam waist for a shorter focal length.

**Figure 7:**
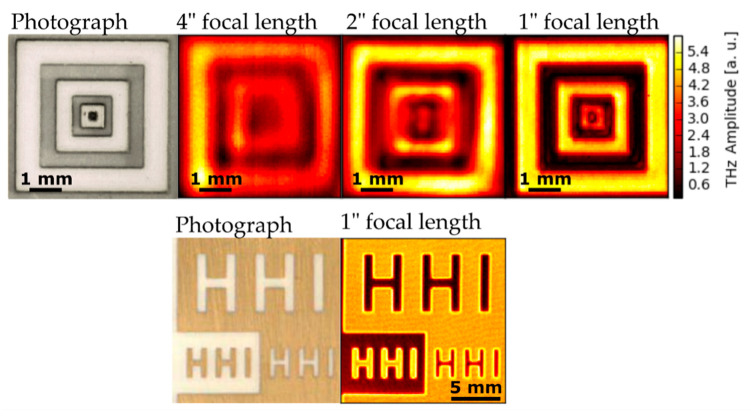
THz imaging experiments with the THz transceiver module. Mirrors with three different focal lengths are compared. Top row: photograph and THz reflection maps of a ceramic sample with a pattern etched into its surface. The reflection maps show the reflected peak-to-peak THz amplitude of the upmost surface. A frequency high pass for the THz spectrum at 1.5 THz was used to limit the spectral range. Bottom row: photograph and reflection amplitude map of a gold pattern on a ceramic sample. This THz image was recorded using the 1” focal length mirror.

In the THz imaging setup, a flat sample is raster scanned with an x-y-stage with a pixel spacing of 50 µm. Full pulse traces with a duration of 70 ps were recorded for each pixel. The sample consists of a ceramic substrate patterned with a relief-like structure (see photograph in Figure 7, top row). The bright squares in the photograph are elevated by 0.5 mm compared to the dark background. The top row of Figure 7 compares the amplitude images for 4″, 2″, and 1″ focal lengths. Note how the resolution of the THz image is improved for parabolic mirrors with shorter focal length. The second row of Figure 7 shows the photograph and THz image of a gold-coated ceramic sample. For all of the shown THz images we used a Bessel type high-pass filter at 1.5 THz, leading to an effective spectral maximum around 1.7 THz. Only with the new InGaAs:Fe based transceiver, which exhibits a spectral maximum around 1.2 THz and significantly increased spectral power for frequency components above 1.5 THz, this high-pass filtering becomes feasible.

The minimum beam waist of a Gaussian beam is closely related to the resolution of an image acquired with it. The beam waist radius *w* behind a lens/mirror is given by the following equation [26]:

(1) $$2w=\frac{4\lambda }{\pi }\frac{f}{D}$$

here, *λ*, *f*, and *D* denote the wavelength, focal length of the mirror, and diameter of the mirror. Thus, the smaller the ratio of focal length to diameter, the smaller the beam waist and the better the lateral resolution. In the reflection head for fiber-coupled THz modules shown in Figure 6a, a parabolic mirror with 1″ diameter and 4″ focal length is used. For this configuration, for the frequencies 0.5, 1, and 2 THz (corresponding to wavelengths of 600, 300, and 150 µm, respectively) beam waist radii of 1.53, 0.76, and 0.38 mm result. However, with a 1″ focal length these values could be improved by a factor of 4 to 380, 180, and 95 µm, respectively. Note that changing the focal length in the reflection head would also change the angle of incidence of the THz beam. Hence, the reflection head would have to be re-designed completely. In contrast, the collinear geometry of the transceiver allows for improving the lateral resolution, simply by changing the focal length of the focusing parabolic mirror.

**Figure 8:**
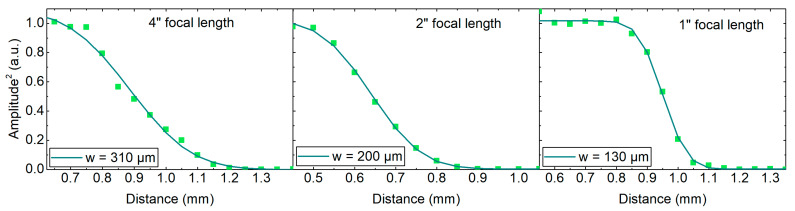
Square of the reflected THz amplitude as a function of distance over an edge of the ceramic sample of Figure 7, top row. The three graphs show the results for the three mirrors with focal lengths of 4″, 2″, and 1″. The data was fitted with the function of Equation (2) and the width of the point spread function *w* was used as a measure of the lateral resolution. For the 4″, 2″, and 1″ focal length mirrors, the extracted values are 310, 200, and 130 µm, respectively.

To extract a quantitative measure of the resolution from the measurements, we analyzed the reflected amplitude as a function of distance at one edge of the ceramic sample, which corresponds to a d-cut through the confocal point spread function. The square of the measured peak-to-peak pulse amplitude as a function of distance is shown in Figure 8 for the three focal lengths used. In order to get a quantitative value for the resolution, we fitted the following function to the data:

(2) $$\left(x\right)=\frac{P_{0}}{2}\left[1-\mathrm{erf}\left(\frac{\sqrt{2}\left(x-x_{0}\right)}{w}\right)\right]+O$$

here, erf(*x*) refers to the error function. *P(x)*, *P_0_*, *x_0_*, *w*, and *O* denote the intensity as a function of distance, maximum intensity, offset in distance, width of the point spread function, and offset in power, respectively. *P_0_*, *x_0_*, *w*, and *O* are fit parameters. Usually, Equation (2) is applied for the knife-edge test of a Gaussian beam and results from the integration over a partially blocked Gaussian intensity profile [27]. Assuming a Gaussian THz beam profile, this equation also applies to the reflected THz power at an edge. Note that *w* is an arbitrary measure of the resolution and does not correspond to the real beam waist of the THz beam. Nevertheless, whatever the resolution criterion is, it can be assumed that it scales similarly to *w*. The fitted values of *w* are 310, 200, and 130 µm (also given in each graph of Figure 8). Hence, the image resolution could be improved by a factor of 2.4 by simply changing the focal length of the focusing mirror from 4″ to 1″. From Equation (1), optimally, one could have expected an improvement by a factor of four in the THz beam waist, translating into an equivalent improvement in resolution. However, the measured confocal point spread function comprises all the deviations from an ideal geometry of both emitter and receiver, reducing the nominal improvement in resolution. The largest error results from the fact that the dimensions of the edge were too small for the largest focal length used to accurately determine the point spread function. Therefore, this value of *w* is not fully comparable to the other two focal lengths. The finite separation between emitter and receiver on the transceiver chip could be an additional source of error. Of course, typical effects of non-ideal optical setups such as cut-off of the beam due to a limited diameter of the mirror are present as well.

Nevertheless, due to its collinear geometry the presented compact, fiber-coupled THz transceiver is a promising tool for high-resolution THz imaging applications.

## 4. Conclusions

We presented a monolithically integrated THz transceiver combining emitter and receiver on a single photoconductive chip based on Fe doped InGaAs. A record high bandwidth of 6.5 THz and a peak dynamic range of 75 dB were obtained for measurements in reflection geometry on a flat mirror. This is a 1.5 THz increase in bandwidth and a 20 dB increase in dynamic range compared to previously reported THz transceivers based on low-temperature-grown InGaAs:Be. The novel InGaAs:Fe transceiver is directly compatible with existing fiber coupled THz TDS systems and allows THz TDS measurements in reflection geometry without the need for a beam splitter or an angled beam path. Furthermore, we compared the fiber coupled transceiver with a reflection head using two separate THz modules as emitter and receiver and an angled THz beam path. With a bandwidth of > 6 THz and a peak dynamic range of 75 dB, the integrated transceiver performs almost as good as the bulky reflection head.

Finally, we demonstrated the advantage of the collinear geometry of the transceiver in a THz imaging experiment. By reducing the focal length of the focusing parabolic mirror, we could increase the numerical aperture of the transceiver and thus improve the lateral resolution of the THz image significantly. With a focal length of 1″ instead of 4″, the image resolution could be improved by a factor of 2.4. Hence, the THz transceiver presented in this paper is a viable alternative to bulky reflection heads using separate emitters and receivers for reflection measurements.

## Figures and Tables

**Table 1:** Summary of the electrical and dynamic properties of different photoconductors that are commonly used as THz antennas. ρ, µ, and τe denote the resistivity, electron Hall mobility, and electron lifetime, respectively. Commercially available THz emitters based on an InGaAs/InAlAs superlattice [12] are abbreviated as SL, THz receivers based on LTG-InGaAs:Be/InAlAs:Be [13] are called LTG. THz antennas based on the InGaAlAs/ErAs material system were presented in [11], where 0.8 mono-layers (ML) of ErAs were deposited in a superlattice.

Material Type	Main Application	n_Doping_ (10^19^ cm^−3^)	n_Hall_ (10^12^ cm^−3^)	ρ (Ω cm)	µ (cm^2^/Vs)	τ_e_ (ps)
SL	TX	Undoped	1.0	2500	2700	40
LTG	RX	0.4	55.0	339	335	0.28
ErAs	TX	0.8 ML	3.6	3850	450	>1
ErAs	RX	0.8 ML	34.4	240	775	0.52
InGaAs:Fe	TX, RX, TRX	50	5.6	1570	710	0.25
